# The relationship between respiratory tract infections caused by toxin-producing bacteria in burn patients during COVID-19: pathogenesis, diagnostics and novel therapies

**DOI:** 10.1099/jmm.0.001997

**Published:** 2025-08-12

**Authors:** Pooriya Hamidniya, Hamid Sedighian, Mahdieh Farzanehpour, Arezoo Fallah, Hamideh Molaee, Mahdieh Mahboobi

**Affiliations:** 1 Applied Virology Research Center, Biomedicine Technologies Institute, Baqiyatallah University of Medical Sciences, Tehran, Iran; 2 Applied Microbiology Research Center, Biomedicine Technologies Institute, Baqiyatallah University of Medical Sciences, Tehran, Iran; 3 Department of Bacteriology and Virology, Faculty of Medicine, Isfahan University of Medical Sciences, Isfahan, Iran; 4 Trauma Research Center, Clinical Sciences Institute, Baqiyatallah University of Medical Sciences, Tehran, Iran

**Keywords:** bacterial toxins, burn units, co-infection, COVID-19, mixed infection, *Pseudomonas aeruginosa*, *Staphylococcus aureus*

## Abstract

The COVID-19 pandemic has significantly increased the complexity of managing burn patients, who are particularly susceptible to bacterial co-infections due to their compromised skin barriers and immune dysregulation. Toxin-producing bacteria, such as *Staphylococcus aureus* and *Pseudomonas aeruginosa*, pose severe risks by producing virulence factors that impair immune function, delay wound healing and exacerbate systemic inflammation. These challenges are amplified in the presence of SARS-CoV-2, as the viral-induced immune dysregulation and cytokine storms worsen clinical outcomes, leading to higher rates of morbidity and mortality. This review explores the interplay between viral and bacterial infections in burn patients during the COVID-19 pandemic, focusing on the role of bacterial toxins, including superantigens from *S. aureus* and exotoxins from *P. aeruginosa* in driving hyperinflammatory responses. These synergistic effects complicate treatment by increasing the likelihood of systemic complications, prolonged hospital stays and MDR infections. To address these challenges, we discuss innovative therapeutic strategies, including endotoxin adsorption therapy to reduce systemic inflammation, immunomodulatory treatments to control cytokine storms and bacteriophage therapy for targeting MDR pathogens. Advanced wound care techniques and rapid diagnostic tools, such as CRISPR-based molecular assays, are highlighted as essential for timely and effective intervention. This review underscores the urgent need for integrated approaches that combine targeted diagnostics, advanced therapeutics and robust infection control measures. These insights aim to improve outcomes for burn patients co-infected with bacterial pathogens and SARS-CoV-2, offering valuable guidance for future pandemic preparedness and burn care protocols.

## Graphical Abstract

Schematic overview of COVID-19–associated bacterial co-infections in burn patients. Burn-related skin barrier damage, immunosuppression, and prolonged hospitalization increase susceptibility to pathogens such as *Staphylococcus aureus* and *Pseudomonas aeruginosa* during SARS-CoV-2 infection.

## Introduction

The COVID-19 pandemic, declared a global health emergency by the World Health Organization (WHO) in 2020, has profoundly disrupted healthcare systems worldwide, placing enormous strain on vulnerable populations, including those with severe burns [1,2]. SARS-CoV-2, the virus responsible for COVID-19, causes a wide range of clinical outcomes, from mild or asymptomatic cases to severe conditions such as acute respiratory distress syndrome (ARDS), septic shock and multiorgan failure [3,4]. Patients with underlying health conditions, including those with extensive burn injuries, are disproportionately affected due to compromised immunity and increased vulnerability to secondary infections [5,6].

Burn wounds compromise the skin’s primary defence barrier, leaving patients highly susceptible to bacterial infections. Burn patients not only experience colonization by opportunistic pathogens but also face the added burden of healthcare-associated infections, particularly during prolonged hospital stays and frequent use of invasive medical devices [7,8]. Secondary bacterial infections are already a significant cause of morbidity and mortality in burn patients, and the COVID-19 pandemic has introduced additional complexities in their clinical management [9,10].

Recent studies indicate that 15–20% of hospitalized COVID-19 patients experience secondary bacterial infections, which significantly worsen clinical outcomes. Patients co-infected with SARS-CoV-2 and bacterial pathogens exhibit higher rates of intensive care unit (ICU) admission, prolonged hospitalizations and elevated mortality risks, particularly in critical care and burn settings [11,13]. Additionally, recent studies confirm that patients co-infected with SARS-CoV-2 and bacterial pathogens face higher rates of ICU admission, prolonged hospitalizations and greater mortality risks, particularly in burn and critical care settings [10,14].

For burn patients, this dual burden of SARS-CoV-2 and bacterial pathogens has created a particularly precarious situation, where the interaction between viral and bacterial infections exacerbates systemic inflammation and immune dysfunction [15].

Multidrug-resistant (MDR) bacteria such as *Staphylococcus aureus* (*S. aureus*), *Pseudomonas aeruginosa* (*P. aeruginosa*), *Acinetobacter baumannii* (*A. baumannii*) and *Klebsiella pneumoniae* (*K. pneumoniae*) pose significant risks for burn patients [16,17]. These pathogens cause severe wound infections and produce virulence factors like potent exotoxins. These toxins, including staphylococcal enterotoxins and *Pseudomonas* exotoxin A, impair the host immune response, leading to tissue damage, delayed wound healing and sepsis [18,19].

Evidence shows that bacterial toxins can modulate immune responses, often exacerbating viral pathogenesis in COVID-19 patients by enhancing viral replication, increasing bacterial colonization and intensifying immune dysregulation [20,21].

During the COVID-19 pandemic, bacterial superantigens, such as toxic shock syndrome toxin-1 (TSST-1) and staphylococcal enterotoxin B from *S. aureus*, have been implicated in worsening cytokine storms, leading to multiorgan damage and increased mortality rates [10]. This superantigen activity has been linked to more severe disease progression in co-infected patients, as it amplifies the immune system’s already heightened response to the viral infection, leading to multiorgan damage and increased mortality [22].

Similarly, *P. aeruginosa* – another major pathogen in burn patients – produces exotoxins such as ExoS and ExoU that impair immune cell function, disrupt epithelial barriers and promote wound biofilm formation [20]. These toxins contribute to local wound infection and have systemic effects that complicate the management of COVID-19 patients, especially when compounded by the effects of viral-mediated immune suppression [23]. Research has shown that bacterial toxins can modulate host-pathogen interactions, increasing viral replication and facilitating bacterial colonization, leading to more severe co-infections [21]. In addition, the immunosuppressive effects of severe COVID-19, particularly in patients who develop ARDS and require mechanical ventilation, can create an ideal environment for bacterial pathogens to thrive [24]. These patients often receive broad-spectrum antibiotics, which can disrupt the balance of the host microbiota and promote the emergence of MDR bacteria, further complicating treatment options [25]. The resulting co-infections, particularly those involving toxin-producing bacteria, are more difficult to manage, often requiring more aggressive antimicrobial therapies and longer hospital stays [25].

New insights further highlight the potential role of bacterial toxins in worsening COVID-19 outcomes through immune modulation and superinfection in patients requiring intensive care and mechanical ventilation, which create ideal conditions for bacterial proliferation [26].

Given these complexities, a deeper understanding of the interactions between SARS-CoV-2 and bacterial pathogens is critical for developing effective therapeutic strategies. This review highlights the challenges posed by bacterial co-infections in burn patients during the COVID-19 pandemic, focusing on the role of bacterial toxins in exacerbating clinical severity. The findings underscore the urgent need for innovative infection control practices, rapid molecular diagnostics and tailored therapeutic approaches to improve outcomes for this vulnerable population.

## Impact of burn severity on infection susceptibility

Burn injuries are classified from first to fourth degree, with the depth of injury correlating to increased infection risk and immune compromise. In particular, severe burns, such as third- and fourth-degree burns, create conditions that make patients highly susceptible to bacterial colonization and systemic infections (Table 1).

**Table 1.** Classification and types of burned wounds [163,166]

Degree	Thickness	Depth	Characteristics	Morphology
First	Superficial	Epidermis	Painful Redness Mild swelling No blister No scar	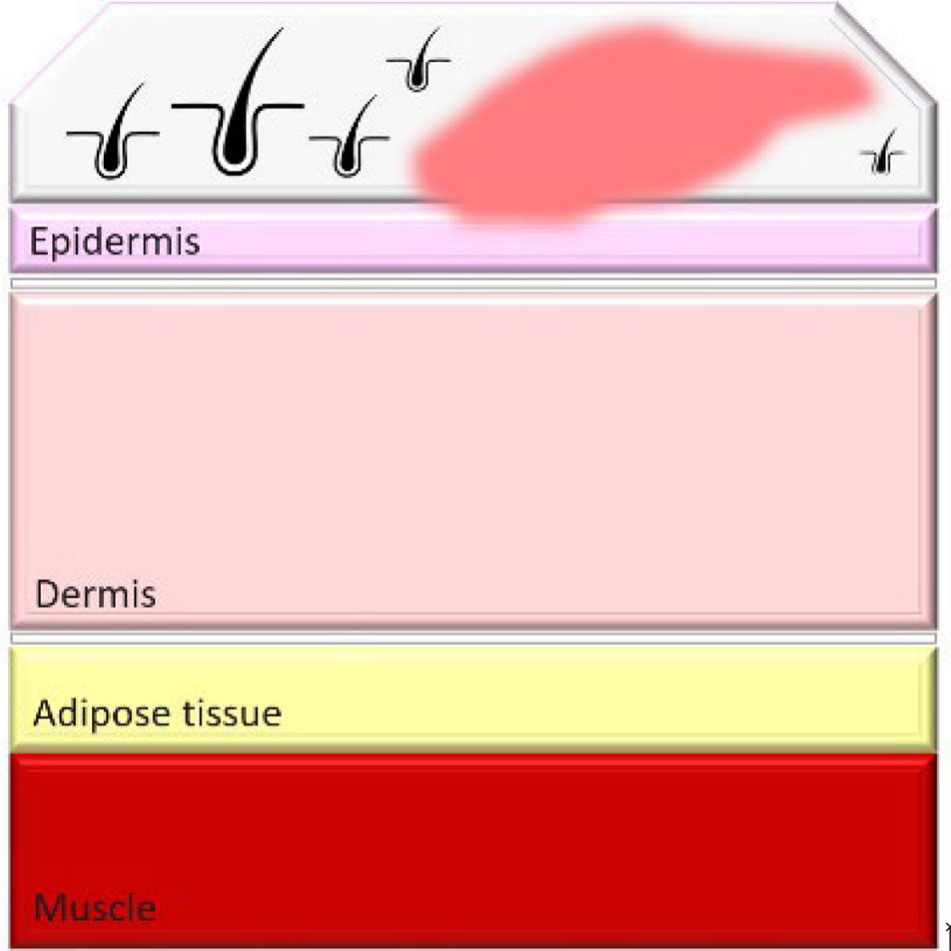
Second	Partial-intermediate	Dermis (papillary region)	Painful Blister and weep Splotchy skin Severe swelling Do not need surgery Scar formation is possible and more painful in superficial-partial thickness Deep partial thickness may lead to surgery, but formed scars are less painful In both partial and intermediate thickness, increased risk of scars and infection is possible with increasing depth	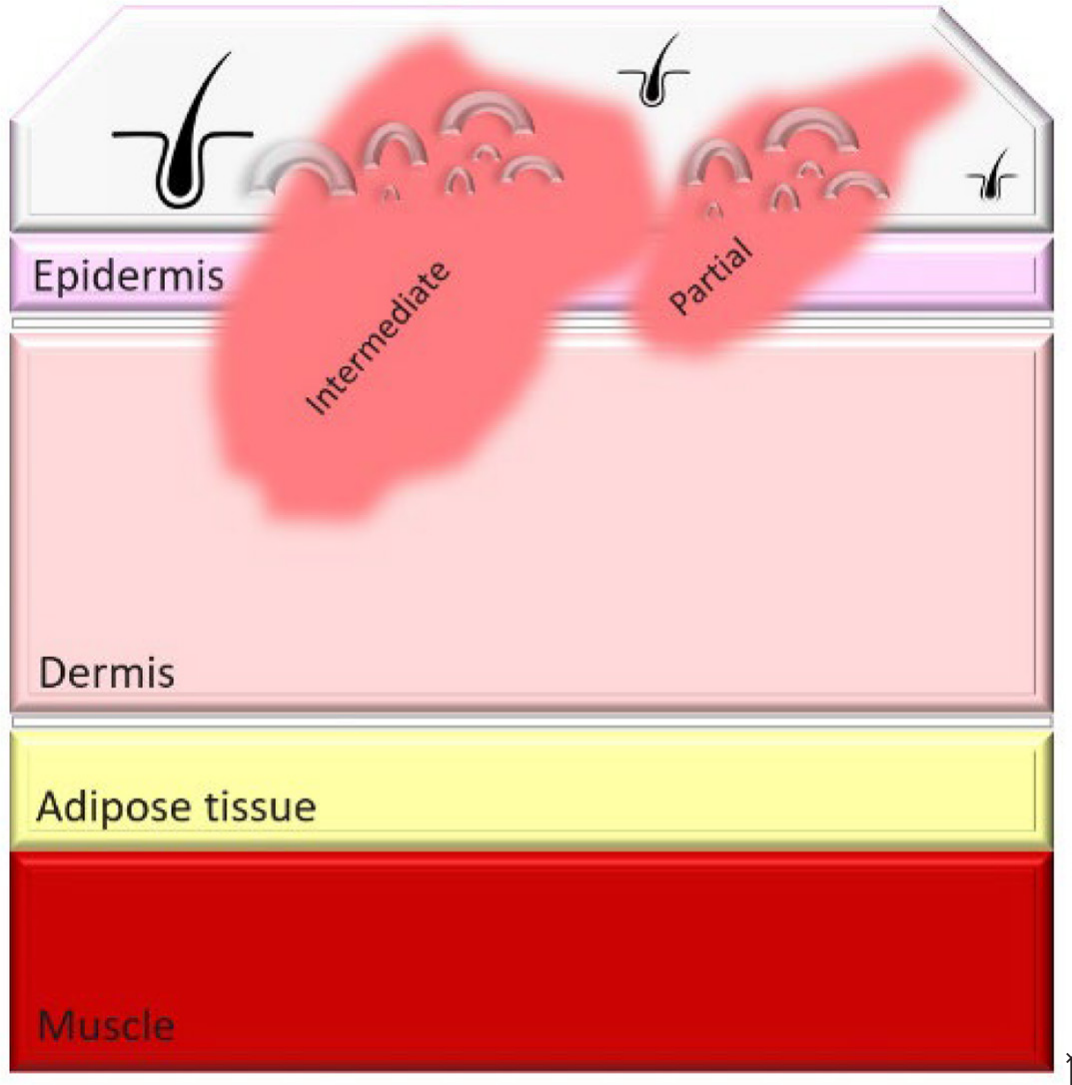
Third	Full-thickness	Dermis (reticular region)	Dry White Leathery Relatively painless Insensate to light touch and stimulation with pin Small tissue parts could heal with substantial scar or contracture Skin grafting may be advised for large skin areas High risk for infection	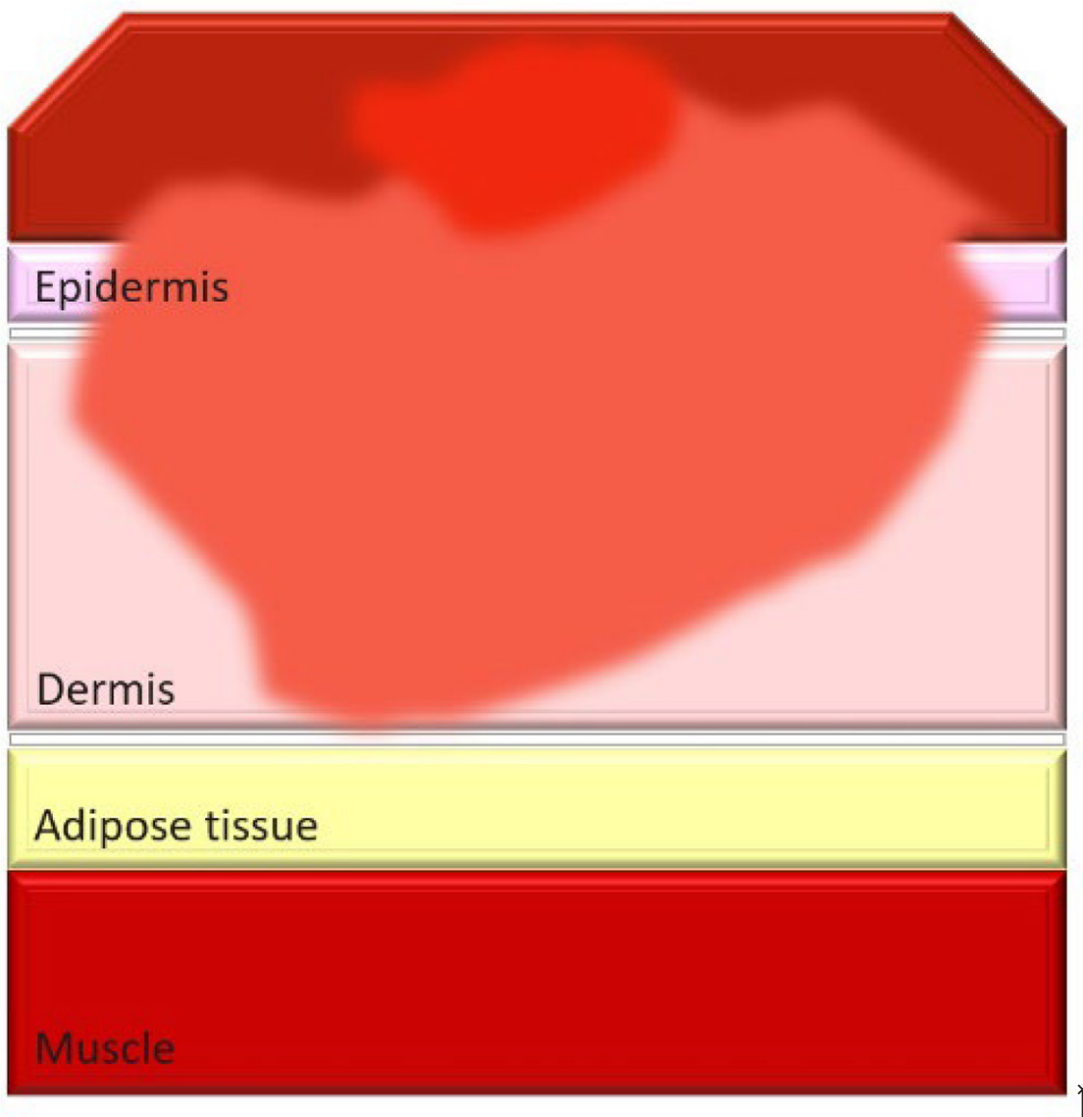
Fourth	Deep full-thickness	Hypodermis (subcutaneous tissue)	Charred scar formation Muscle and bone are involved Insensate Increase the risk for the loss of burned part	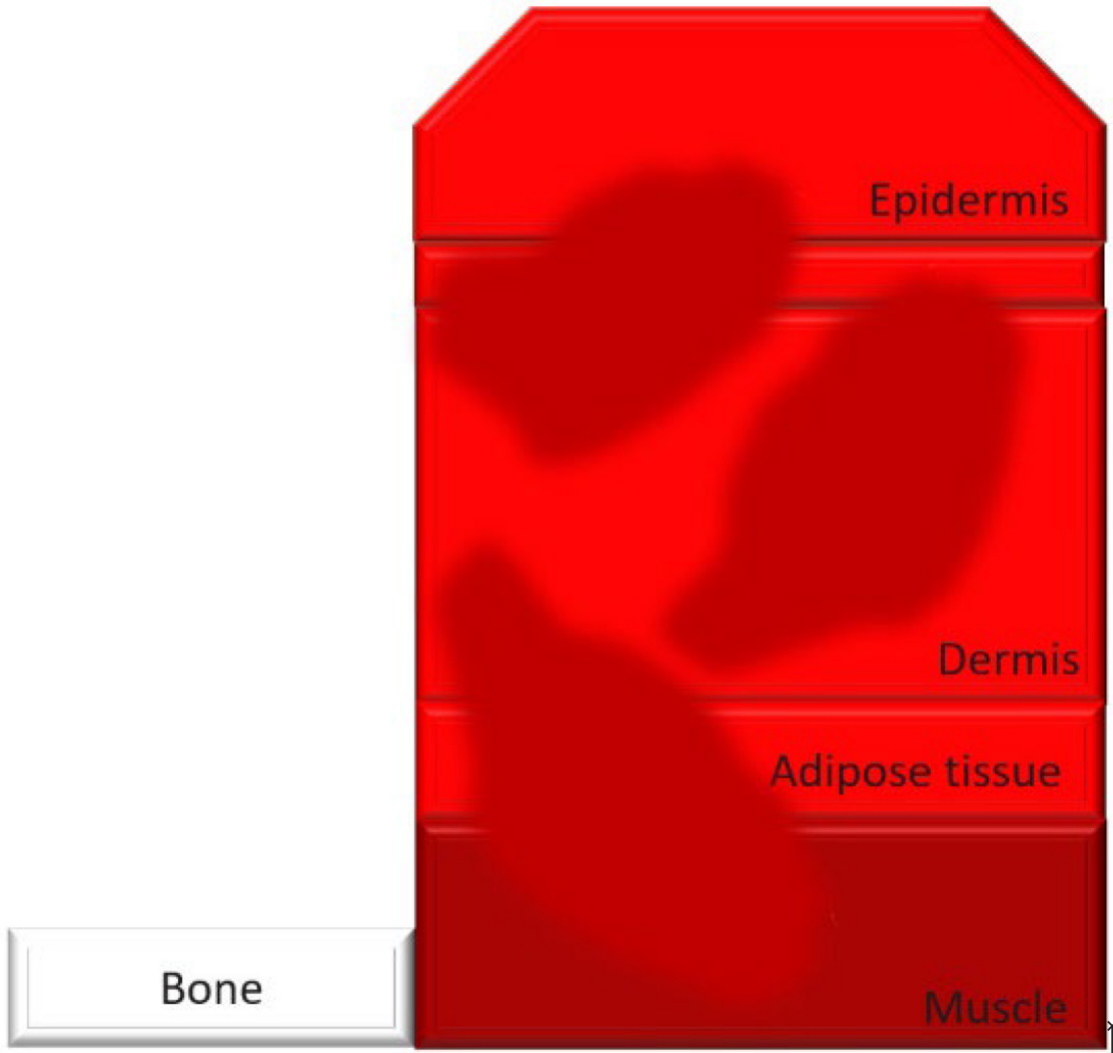

### Weakness-thickness burns (first degree)

First-degree burns, also known as superficial burns, are the mildest form of burn injury, affecting only the outermost layer of skin, the epidermis. These burns typically present with redness, mild swelling and pain but do not blister, as the damage is confined to the skin’s surface. Common causes include brief contact with hot objects, mild sunburn or scalding from hot liquids. First-degree burns generally heal on their own within 3–7 days without leaving scars, as the skin barrier remains largely intact and regenerative processes quickly restore damaged tissue. Since there is no disruption to the deeper dermal layers, first-degree burns pose minimal risk of infection, provided the skin remains unbroken. Proper care, such as maintaining cleanliness and applying soothing topical treatments, can reduce discomfort and support healing [27]. The typical immune response to first-degree burns includes local inflammation, which helps prevent infections by recruiting immune cells to the site of injury. Maintaining skin hydration and using topical agents like aloe vera or hydrocortisone can further support the healing process and prevent complications [28].

### Partial-thickness burns (second degree)

Second-degree burns involve damage to the dermis and can be either superficial or deep. Superficial second-degree burns are typically painful and blistered but heal with minimal scarring if no infection occurs. Deep second-degree burns, however, can disrupt skin integrity enough to increase infection risk, especially when wound care is inadequate. Proper management is essential to prevent local infections that may extend deeper, complicating healing and potentially leading to systemic infections [29,30]. The clinical management of second-degree burns includes cleaning the wound, applying appropriate dressings and monitoring for signs of infection such as increased redness, swelling or discharge. Superficial second-degree burns heal with minimal intervention, whilst deep burns may require debridement and more intensive care to prevent infection [31]. Signs of infection in second-degree burns include increased pain, pus formation and fever. Prompt treatment with topical or systemic antibiotics can help manage these infections effectively [32].

### Full-thickness burns (third degree)

Third-degree burns, also known as full-thickness burns, destroy both the epidermal and dermal layers of the skin, resulting in dry, leathery and insensate areas due to nerve damage. With all protective skin barriers lost, third-degree burns are highly susceptible to bacterial colonization. The damaged tissue provides an ideal environment for bacterial growth, and the absence of sensation in these areas complicates early infection detection. Infections in third-degree burns can progress rapidly, leading to sepsis, which further weakens immune defences and complicates treatment [27]. The immune response to third-degree burns is significantly impaired, making it difficult for the body to combat infections. Advanced treatment options include the use of skin grafts, advanced wound dressings with antimicrobial properties and systemic antibiotics to control and prevent infections [33]. Infections can severely impact the recovery process, leading to longer hospital stays, increased need for surgical interventions and higher mortality rates [34].

### Deep full-thickness (fourth degree)

Fourth-degree burns extend through the skin into muscles, tendons and bones. This degree of injury not only removes surface defences but also initiates systemic inflammation, making patients extremely vulnerable to bacterial co-infections. In fourth-degree burns, immune suppression is particularly severe, as necrotic tissue serves as a reservoir for bacterial pathogens. Under pandemic conditions, the risk of bacterial co-infection is further heightened, as co-infection with SARS-CoV-2 can exacerbate immune dysfunction, leading to worse clinical outcomes [35]. The systemic impact of fourth-degree burns includes widespread inflammation, immune suppression and increased susceptibility to infections. Managing these burns requires a multidisciplinary approach, involving infectious disease specialists, surgeons and critical care teams to address the complex needs of these patients [36]. Recent research indicates that bacterial toxins from MDR bacteria can significantly worsen outcomes in patients with severe burns and SARS-CoV-2 co-infections, emphasizing the need for aggressive antimicrobial therapies and innovative treatment strategies [37].

## Types of burns and infection risk

Burn injuries may result from thermal, electrical, chemical or radiation exposure, each presenting unique challenges for treatment and infection management. Additionally, non-accidental causes account for 3–10% of burns in children, adding a layer of complexity in paediatric burn care [38,39].

### Thermal burns

Approximately 70% of burns in children result from scalding, often caused by hot liquids, hot water exposure in showers or spilled drinks. Elderly individuals are also susceptible to scald burns due to reduced mobility or sensory deficits. These burns are usually first- or second-degree and are prone to infection if the skin barrier is compromised, requiring careful wound management to prevent bacterial colonization [40,41]. Proper initial care involves cooling the burn with running water, avoiding ice and covering the burn with a sterile, non-adhesive bandage. Monitoring for signs of infection, such as increased redness, swelling or discharge, is crucial, as is maintaining hygiene to prevent bacterial colonization [42].

### Flame burns

Up to 50% of burns in adults are caused by flame exposure, frequently leading to deeper injuries, such as severe second- and third-degree burns. Flame burns are often accompanied by inhalation injuries and other trauma, creating conditions for complex infections. The depth of injury in these burns often results in damaged tissue that can harbour bacterial pathogens, especially under compromised immune conditions [43,44]. Immediate treatment includes stopping the burning process by removing the individual from the source of the fire and using cool water to reduce the burn’s temperature. Inhalation injuries should be assessed, and patients may require supplemental oxygen or intubation. The risk of infection is mitigated by debridement of necrotic tissue, application of antimicrobial dressings and systemic antibiotics when necessary [45].

### Electrical burns

Deaths from electrical injuries are increasing globally, particularly amongst men aged 20–40 years. Electrical burns, which account for 20% of burn-related fatalities, can cause damage through direct contact, electric current transmission or thermal effects. These injuries vary widely; whilst contact with low-voltage sources like household outlets rarely results in fatality, high-voltage exposures can cause deep tissue injury that isn’t immediately visible on the skin. This hidden damage raises the risk of deep infections and systemic complications, often requiring intensive care and debridement [46,47]. Initial assessment of electrical burns includes evaluating the entry and exit wounds, which can help determine the extent of internal damage. Cardiac monitoring is essential due to the risk of arrhythmias. Burn care involves debridement, monitoring for compartment syndrome and aggressive infection control measures, including the use of prophylactic antibiotics when deep tissue injury is suspected [48].

### Chemical burns

Chemical burns, which represent 3–6% of all burns, have a relatively high mortality rate of 14–30% due to the severity of tissue damage. Commonly resulting from industrial accidents or exposure to household chemicals, chemical burns typically occur after contact with strong acids or alkalis. Unlike thermal burns, chemical burns involve prolonged tissue exposure, leading to delayed tissue necrosis. Because tissue damage may not be immediately apparent, these burns require close monitoring for infection as the extent of injury unfolds over several days [49,50]. Immediate treatment involves removing contaminated clothing and flushing the area with copious amounts of water to dilute the chemical. Continuous irrigation is crucial to prevent further tissue damage. Infection risk is high, especially if initial wound care is delayed. The use of topical antimicrobial agents and systemic antibiotics can help manage and prevent infections [51].

## Infection risks in burn patients during the COVID-19 pandemic

Burn patients already face considerable infection risks associated with each burn type, which complicates patient care – especially in the presence of immune suppression and the prevalence of hospital-acquired infections. With the onset of the COVID-19 pandemic, managing these patients has become even more challenging. Co-infections with SARS-CoV-2 increase susceptibility to bacterial pathogens and intensify immune responses, leading to a heightened risk of severe complications. This overlap between burn wound infection risk and COVID-19-associated challenges necessitates a closer examination of how the pandemic has specifically impacted outcomes for burn patients [52,53].

### Burn wounds and COVID-19

Since burn patients often require extended hospitalizations and multiple surgeries, their exposure to infection and risk of COVID-19 transmission has increased during the pandemic. As a result, it has become necessary to evaluate changes in healthcare practices and the epidemiological characteristics of burn patients in response to COVID-19 [54].

At the pandemic’s onset, strict guidelines were implemented to minimize COVID-19 transmission amongst burn patients. These guidelines included measures such as reducing the number of in-person visits, implementing telemedicine for consultations and prioritizing urgent and emergency procedures. Non-essential procedures were postponed, and protective measures for healthcare personnel were prioritized based on their contact level with patients [55]. Additionally, patients from high-prevalence areas were advised to seek treatment locally rather than travelling to other provinces, minimizing potential virus spread. Teleconsultations with experienced burn specialists were encouraged to reduce unnecessary movement and exposure [56]. Burn severity and patient assessments were conducted remotely whenever possible, relying on telemedicine to reduce in-person interactions. Patients from high-prevalence areas were advised to seek treatment locally rather than travelling to other provinces, and teleconsultations with experienced burn specialists were encouraged to minimize unnecessary movement. Screening for COVID-19 symptoms or history of the disease was mandatory for all incoming burn patients, and those with COVID-19 co-infection were quarantined in designated hospital areas with strict adherence to contact protocols [57,59]. This helped in creating a controlled environment where both burn care and COVID-19 precautions could be managed simultaneously.

Studies indicate that although overall burn-related hospitalizations did not increase during quarantine, burn injuries were often more severe, involving larger body surface areas. These more extensive injuries have placed additional strain on healthcare systems already burdened by pandemic-related demands [60]. Public health messages should prioritize burn prevention strategies during quarantine and isolation periods to reduce injury incidence and ease the strain on healthcare resources [61,62]. It is crucial to emphasize the importance of home safety measures, such as keeping hot liquids out of children’s reach and ensuring safe cooking practices to prevent accidental burns.

**Fig. 1.**
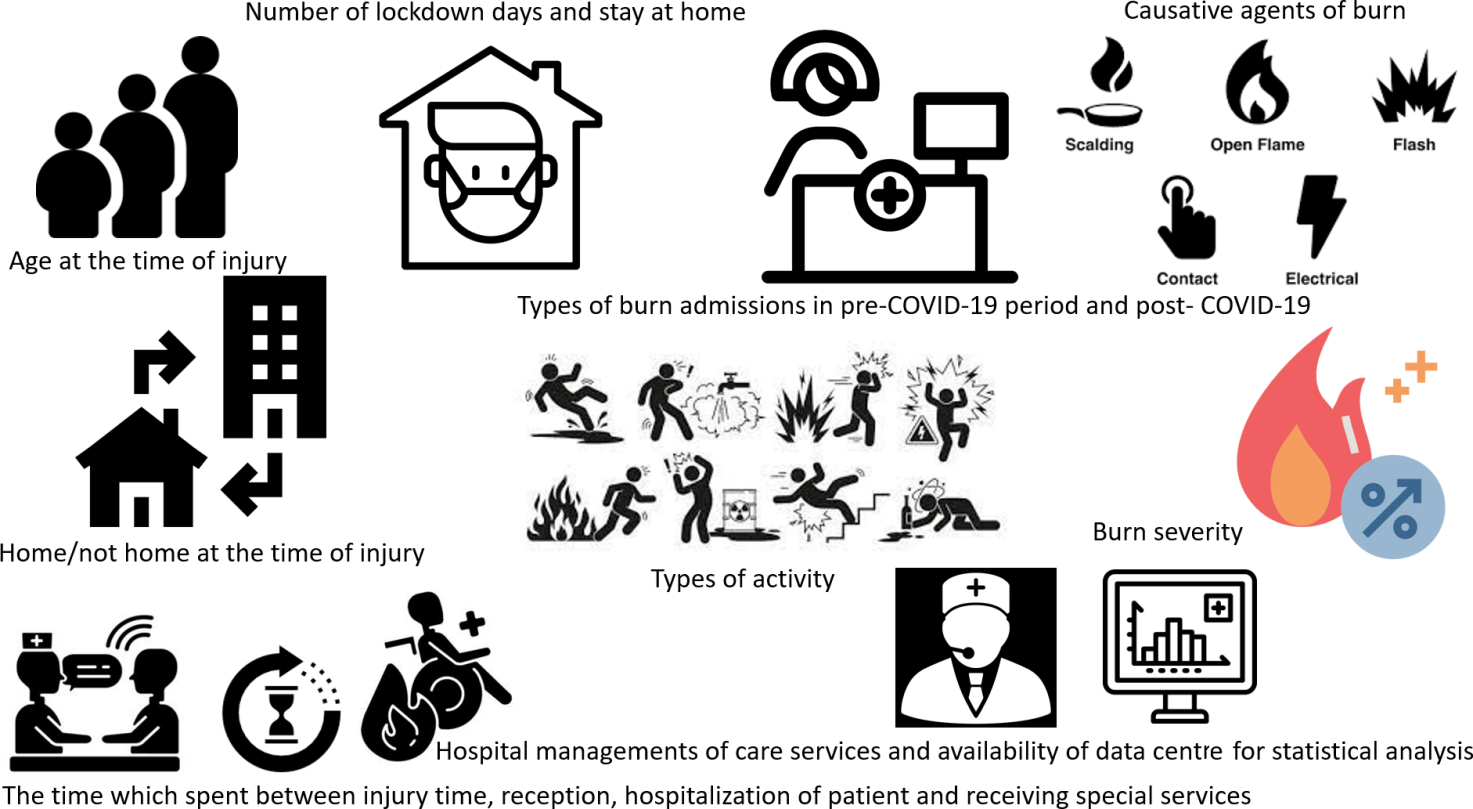
Variables affecting the statistics and analysis of burns in the era before and after the pandemic.

The different variables impacting the statistical analysis of burn-care systems during the COVID-19 pandemic are shown in Fig. 1. These variables include the number of burn admissions, the severity of burns, infection rates and the outcomes of patients with and without COVID-19 co-infection.

### Thrombotic and wound healing complications

Angiotensin-converting enzyme II (ACEII) is widely expressed on the endothelium lining of blood arteries and is a major entrance site for the SARS-CoV-2 virus. Once inside, the cell is easily accessible, which causes the virus to multiply, lyse and reveal the prothrombotic basement membrane. Similarly, viral binding leads to ACEII internalization, which raises angiotensin II plasma levels. Vasoconstriction and a pro-thrombotic, pro-inflammatory condition are facilitated by elevated angiotensin II. This activity further strengthens the pro-thrombotic condition by raising circulating quantities of interleukin (IL)-6. Notably, SARS-CoV-2 can also attach to and activate the TACE/ADAM17 enzyme, which converts TNF-α. The rise in active circulating TNF-α is caused by this enzyme. These serve as chemo-attractants for monocytes and macrophages when combined with IL-6 and other activated cytokines. Due to the overwhelming activation of immune cells within the vessel wall, the result is localized vasculitis. Unsurprisingly, compared to their matched counterparts, burned individuals with COVID-19 were 2.641 times more likely to experience thrombotic problems. Although the physiology of burn patients has not been demonstrated to be impacted by ACEII function, there is a notable overlap in TNF-α and IL-6 activity. These elements, along with the virus’s destruction to the epithelium, could counteract any antithrombotic effects that the burn patient may be experiencing [37]. Furthermore, the COVID-19-induced hypercoagulable state may further impair blood flow to the burn wound, postponing healing and raising the risk of infection. Additionally, burned individuals may require many treatments and may have mobility issues. Not surprisingly, the majority of burned patients meet Virchow’s triad of endothelial damage, hypercoagulability and stasis. Because of these factors, we anticipate a higher incidence of thrombotic problems [37].

### Immunopathology and secondary infections

Inflammatory cytokines and chemokines, including IL-1, IL-6, IL-8, IL-17, CCL-2, TNF-α, G-CSF, IP-10, MCP-1 and MIP, are elevated in COVID-19 patients. These markers' concentrations vary based on the health of the individual; elevated cytokine levels, particularly IL-6, appear to be directly linked to a worsening of the patient’s condition [63]. Furthermore, increased cytokine levels could be regarded as prognostic markers in the clinic because they quickly cause a patient’s condition to worsen and ultimately result in death [64].

In summary, it appears that innate immunity, which is represented by neutrophils and proinflammatory cytokines in the form of a cytokine storm, is attempting to contain the infection and defeat the virus, but instead of doing so, it causes an overwhelming inflammatory response. This results in lung damage with tissue damage and potentially fatal oedema that is loaded with mucins and fibrin, which regrettably leads to severe respiratory failure and mortality in patients who are significantly impacted [65].

This has translational implications and is significant for burn care practitioners. Damaged tissues in the context of a significant burn injury trigger a systemic and local inflammatory response by mechanisms akin to those of COVID-19. Accordingly, patients with COVID-19 who have burn injuries may react more strongly to the burn insult due to their pre-existing hyperinflammatory and hypercoagulable conditions [66].

In burn patients infected with SARS-CoV-2, subepidermal bullae contain red blood cells and a few white blood cells. The dermis showed granulation tissue with prominent lymphocyte infiltration. The subepidermal bulla with underlying lymphocyte-rich granulation tissue was also seen. Also, in another case, subepidermal blisters containing red blood cells and a few lymphocytes and rarely polymorphonuclear leukocytes (PMN) leukocytes were present. The dermis showed infiltration of lymphocytes and a few PMN leukocytes as well as increased fibroblasts and small vessels (like granulation tissue). Some fragments of scar tissue and lymphocyte-rich granulation tissue were seen in the underlying dermis in COVID-19 patients [67].

Patients with COVID-19 who present with burn injuries require careful attention and risk/benefit analysis, including a pre-operative assessment of respiratory function and systemic inflammation. To properly prevent and/or treat COVID-19-related hyperinflammatory consequences, such as hypercytokinemic response and coagulopathy, more investigation is needed to clarify and validate the pathogenesis [68,70].

### Immunomodulatory approaches in burn patients during COVID-19

The complexity of immune responses in burn patients co-infected with SARS-CoV-2 presents significant clinical challenges, especially given the heightened risk of cytokine storms and immune dysregulation. Cytokine storms, characterized by an excessive release of pro-inflammatory cytokines, can lead to severe inflammation and organ damage. Managing this hyperinflammatory response whilst maintaining essential immune functions is critical [66,71].

Immunomodulatory therapies have gained interest as potential supportive treatments for these patients, focusing on reducing hyperinflammation whilst maintaining essential immune responses. In burn patients, who already experience trauma-induced immune suppression, COVID-19-related immune dysregulation can further complicate infection management and healing processes [72].

Corticosteroids, for instance, have been used selectively in COVID-19 patients to moderate cytokine storms by dampening excessive inflammatory responses. The use of corticosteroids like dexamethasone has been shown to reduce mortality in severely ill COVID-19 patients by decreasing inflammation and preventing respiratory complications [73,74]. However, their use in burn patients must be carefully balanced to avoid impairing wound healing and increasing the risk of secondary infections [75]. Studies have shown that low-to-moderate corticosteroid doses can be effective in reducing COVID-19 severity in critically ill patients, but more research is needed to determine optimal dosing and duration for burn patients [76].

Another promising approach involves IL-6 inhibitors, such as tocilizumab, which target cytokine pathways involved in hyperinflammation. Tocilizumab has been effective in reducing the progression of severe respiratory symptoms in COVID-19 patients by blocking IL-6 receptors and minimizing systemic inflammation. Early data suggest that IL-6 inhibitors may improve outcomes in COVID-19 patients by reducing respiratory complications and potentially aiding recovery in co-infected burn patients by minimizing immune system overactivation [77,78].

Janus kinase (JAK) inhibitors, including baricitinib, are another class of immunomodulatory agents under investigation. These drugs work by inhibiting JAK pathways, which are involved in cytokine signalling. Baricitinib has shown potential in reducing inflammation and preventing cytokine storms in COVID-19 patients by interrupting key signalling pathways that drive the hyperinflammatory response. They may offer similar benefits in burn patients with COVID-19, though studies specific to burn populations are still needed [79,80].

Incorporating these therapies into burn care protocols during the COVID-19 pandemic requires a balanced approach, as immunomodulation must be carefully tailored to avoid exacerbating wound infections or delaying wound healing. Continued research on the safety and efficacy of immunomodulatory treatments in burn patients co-infected with SARS-CoV-2 could provide critical insights, ultimately helping to improve outcomes for this vulnerable population [81,83]. This research will help determine the best practices for managing the dual challenges of severe burns and COVID-19 co-infection, ensuring that patients receive the most effective and safe treatments available.

## Increased complexity of infections in burn care during COVID-19

The COVID-19 pandemic has intensified the challenges of managing infections in burn patients. Not only are these patients more vulnerable to healthcare-associated infections, but the pandemic has increased the prevalence of mixed infections, where viral and bacterial pathogens coexist. In burn wounds, bacterial toxins further complicate the immune response, often leading to severe outcomes. This complex interaction underscores the need to understand the impact of infections and microbial toxins in burn patients, particularly during COVID-19, when immune compromise is often heightened [84,85].

### Infection, mixed infection and microbial toxins in burn wounds, especially during the COVID-19 pandemic

**Fig. 2.**
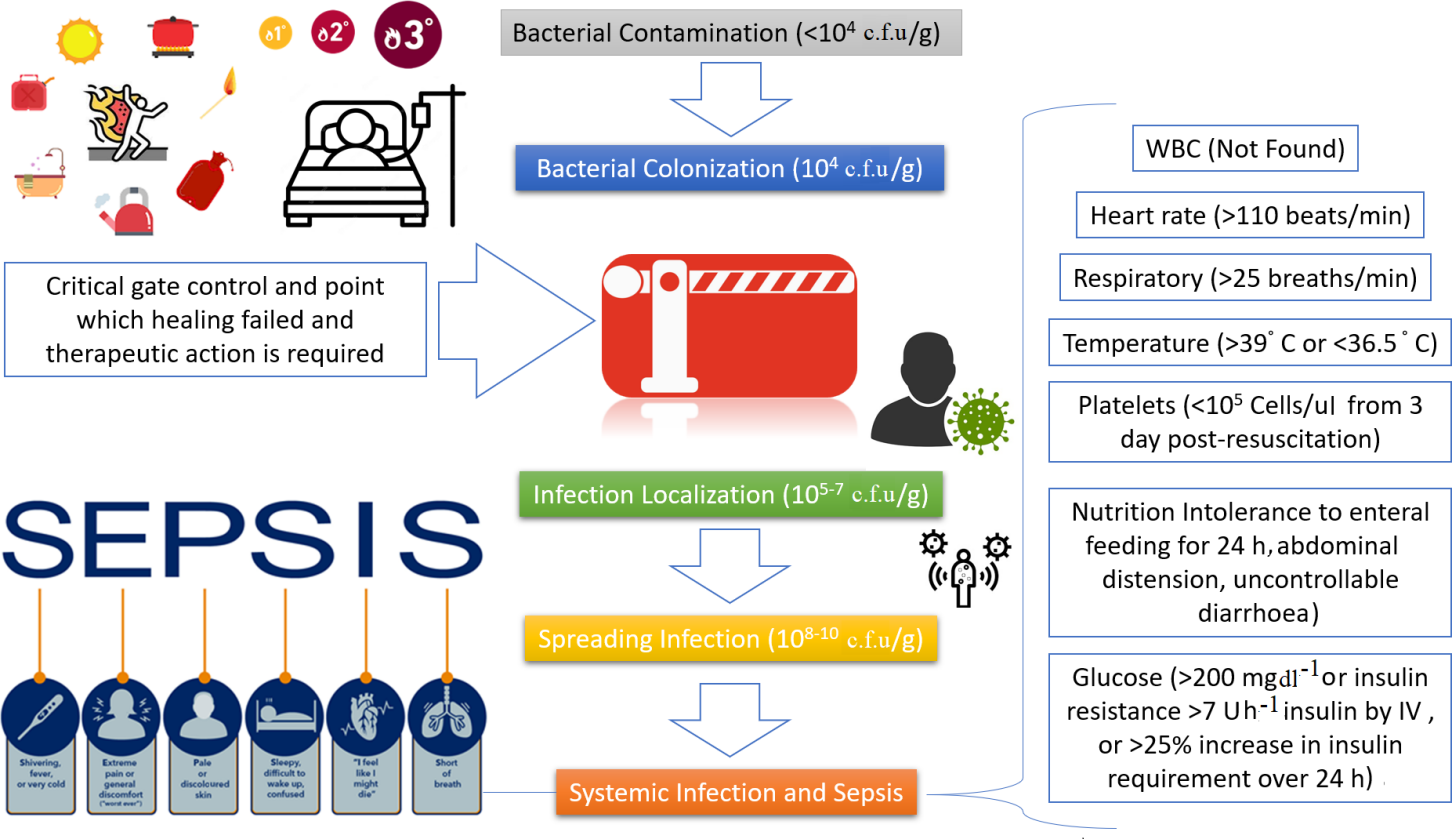
Correlation of wound’s bacterial load and criteria of American Burn Association guideline for sepsis: three or more burn-specific systemic inflammatory response syndrome and expanded parameters with an identified or suspected infection could indicate sepsis in a burn patient. IV, intra-venous.

Healthy skin is essential for maintaining fluid balance, regulating temperature and protecting against infections. It also supports immune function, sensory regulation and vitamin D synthesis [86]. Burns, however, severely damage the skin and impair these functions [87]. This makes burn patients particularly vulnerable to infections, and detecting infections in smaller burns can be challenging due to pre-existing redness and warmth in the affected area. Common infection indicators in burn wounds include colour changes, bruising, swelling, burn deepening, greenish/blue discharge, dehydration and fever. Advanced infections may lead to sepsis or toxic shock, presenting with dizziness, high fever and vomiting (Fig. 2) [58]. Burn wound infections can be caused by a variety of micro-organisms, including fungi, viruses or bacteria, with multiple pathogens often involved [88]. Bacterial infections are the most common, with bacteria entering the wound either through self-inoculation (from the patient’s own flora) or hospital sources. Self-inoculation might occur through contact with faecal flora, whereas hospital-acquired infections typically arise from poor aseptic techniques during dressing changes or contaminated environmental sources, like ventilation systems [61]. The presence of MDR organisms adds to the complexity, requiring meticulous infection control practices and tailored antimicrobial therapies.

Multiple factors influence infection treatment outcomes in burn patients, including age, gender, seasonal conditions, length of hospitalization, burn characteristics and the bacteria involved along with their resistance profiles [89]. Effective infection management requires close antimicrobial monitoring, antiseptic wound care, early excision of necrotic tissue, proper wound dressing, sufficient nutrition and the careful management of shock and resuscitation [90]. Additionally, the use of advanced diagnostic tools can aid in the early detection and treatment of infections, potentially improving outcomes.

MDR organisms like *S. aureus*, *P. aeruginosa*, *Klebsiella*, *Enterococcus*, *Escherichia coli*, *Acinetobacter* and *Enterobacter* pose significant challenges in burn infection treatment, complicating clinical care and patient outcomes [91]. During the COVID-19 pandemic, burn patients faced additional infection-related challenges due to co-infections with SARS-CoV-2 and bacterial pathogens. Bacteria release virulence factors, including toxins, that are central to their pathogenesis [92]. These virulence factors can include enzymes, exotoxins and endotoxins that disrupt host cellular functions and immune responses.

The impact of these toxins varies based on factors such as bacterial load, patient immune response and quality of medical care (Table 2) [93]. COVID-19’s immune dysregulation amplifies the effects of bacterial toxins, complicating both diagnosis and treatment in burn patients and leading to worsened outcomes [94]. For instance, bacterial superantigens can exacerbate cytokine release, intensifying inflammatory responses and contributing to severe complications like toxic shock syndrome and multi-organ failure [95]. Understanding the synergistic effects of viral and bacterial co-infections in burn patients is crucial for developing comprehensive treatment strategies. This includes not only managing the acute infections but also addressing the long-term impact on immune function and overall health outcomes.

**Table 2.** Most important bacterial toxins during mixed infection

Class of bacterial toxins	Target of toxin	Bacteria	Toxin	Reference
Toxins which act on the cell surface	Immune cells (superantigen group toxins)	*S. aureus* *Streptococcus pyogenes*	SEA-SEI, TSST-1, SPEA, SPEC, SPEL, SPEM, SSA, SMEZ, SPEB, ETA, ETB and ETD	[167,168]
	Surface molecules	*P. aeruginosa*	Aminopeptidase	[169]
	Cell membrane	*Bacteroides fragilis*	BFT enterotoxin	[170]
	Large pore formers	*Streptococcus pneumoniae*	Pneumolysin	[171]
	Small pore formers	*S. aureus*	γ-Haemolysins (HlgA-HlgB and HlgC-HlgB), α-toxin and PVL leukocidin (LukS-LukF)	[135,172]
	RTX toxins	*E. coli*	HlyA	[173]
	Membrane perturbing	*S. aureus*	δ-Haemolysin	[174]
	Insecticidal toxins	*E.coli*	HlyE	[175]
Toxins which act on intracellular targets	Protein synthesis	*P. aeruginosa*	PAETA	[176]
	Signal transduction	*E. coli*	LT, cytotoxin necrotizing factors 1 and 2 (CNF1, 2)	[177]
	Cytoskeleton structure	*E. coli*, *Clostridium perfringens*	Lymphostatin, iota toxin and related proteins	[178]
	Intracellular trafficking	*S. pyogenes*	NAD glycohydrolase	[179]
Toxins which are injected into the cells	Mediators of apoptosis	*Salmonella* and *Shigella*	SipB and IpaB	[180]
	Inositol phosphate metabolism	*Salmonella* species	SopB	[181]
	Signal transduction	*P. aeruginosa*, *S. aureus*	ExoS, EDIN-A, B and C	[182]
Bacterial lipopolysaccharides (LPS)	Causative agents of endotoxemia and multi-organ failure (MOF)	*P. aeruginosa*, *Acinetobacter*, *Klebsiella* and *E. coli*	Endotoxins	[183]

### Mixed infections and complications from toxin-producing bacteria in burn patients

As infections in burn patients become more complex, managing mixed infections and toxin-related complications is essential, especially during the COVID-19 pandemic. Burn patients are highly susceptible to mixed infections due to compromised skin integrity and immune dysfunction. Hospital-acquired infections with Gram-negative bacteria like *E. coli*, *P. aeruginosa* and *Acinetobacter* spp. are particularly common in these patients [96]. During the COVID-19 pandemic, infections from toxin-producing bacteria often worsened clinical symptoms, with bacterial and viral symptoms sometimes overlapping and complicating pathogen identification [58]. For instance, *S. aureus* can trigger a cytokine storm similar to the inflammatory response in severe COVID-19. Superantigen-like toxins (SSLs) from *S. aureus* induce widespread immune activation, which poses a heightened risk for burn patients with pre-existing immune dysregulation from trauma or infection [97]. Studies show that SSLs can exacerbate immune activation, often leading to systemic complications in burn patients [64], and this is especially relevant as dysregulated immune responses in both severe burns and COVID-19 can have life-threatening consequences [98]. Research indicates that superantigens, such as TSST-1 from *S. aureus*, are potent immune activators, driving systemic inflammation in vulnerable populations like burn patients. Superantigens work by cross-linking MHC on antigen-presenting cells with T-cell receptors, causing non-specific T-cell activation and the release of cytokines like TNF-α, IL-1β and IL-6 [99]. This mechanism contributes to a hyperinflammatory state akin to the cytokine storm in severe COVID-19, illustrating how bacterial superantigens may worsen viral pathogenesis [100]. This is of particular concern for burn patients, who are at higher risk of severe complications due to immune dysregulation from both trauma and infection [101]. Infections involving toxin-producing bacteria like *S. aureus* and *P. aeruginosa* add complexity. These pathogens release exotoxins that damage tissue and trigger systemic immune responses. Recent studies show that in burn patients co-infected with COVID-19, exotoxins further dysregulate the immune response, leading to poorer outcomes [10]. For instance, exotoxin A from *P. aeruginosa* and staphylococcal enterotoxins delay wound healing, promote biofilm formation and heighten inflammation, particularly in burn patients also infected with SARS-CoV-2. Exotoxin A inhibits protein synthesis in host cells, leading to cell death and impairing wound healing by disrupting re-epithelialization and granulation tissue formation. In patients with concurrent COVID-19, this is compounded by cytokine storms induced by the virus, which amplify systemic inflammation and compromise immune defences [4,102]. The presence of staphylococcal enterotoxins further complicates the situation by acting as superantigens, which non-specifically activate T-cells and lead to a significant release of pro-inflammatory cytokines, potentially intensifying COVID-19-related respiratory symptoms [10,102, 103]. Both *P. aeruginosa* and *S. aureus* can form biofilms in chronic wounds, creating a barrier that hinders antibiotic effectiveness and sustains inflammation. This is especially problematic in COVID-19, where combined viral and bacterial inflammatory responses increase risks for pneumonia and ARDS, worsening outcomes [10,14, 96].

### Gram-negative bacteria and endotoxin-mediated complications

Hospital-acquired infections with Gram-negative bacteria, such as *E. coli*, *P. aeruginosa* and *Acinetobacte*r spp., are common in burn patients [96]. These pathogens release endotoxins [lipopolysaccharides (LPS)], which induce strong systemic inflammatory responses. In COVID-19-positive burn patients, endotoxemia – characterized by high circulating endotoxin levels – can progress to septic shock or multi-organ failure [104]. Studies show that antibiotic-induced endotoxin release during treatment can worsen outcomes by triggering heightened immune responses, already elevated due to SARS-CoV-2 [105,107]. Burn patients with COVID-19 often require broad-spectrum antibiotics to manage secondary bacterial infections. This antibiotic use can lead to the massive release of endotoxins from lysed Gram-negative bacteria, exacerbating the patient’s condition [108,109]. Research demonstrates that antibiotic therapy in burn patients significantly increases circulating endotoxin levels, contributing to systemic inflammatory response syndrome and poor clinical outcomes [66,110, 111]. In COVID-19 patients with immune dysregulation, endotoxemia can trigger inflammatory responses that amplify viral pathogenesis [97].

### Endotoxemia and endotoxin-like complications in COVID-19 and burn patients

Endotoxemia has emerged as a significant complication during the COVID-19 pandemic, even in patients without Gram-negative bacteremia. Studies suggest that this may be linked to factors such as acute kidney injury (AKI), a common issue in severe COVID-19 cases. AKI impairs the body’s ability to clear endotoxins, leading to their accumulation, which in turn causes inflammation and endothelial damage, complicating the clinical course [112,114]. This highlights the complexity of endotoxemia in viral infections, where bacterial byproducts like LPS may exacerbate disease severity in the absence of active bacterial infections. Furthermore, systemic inflammation from viral infections can compromise the intestinal barrier, potentially allowing the translocation of bacterial endotoxins into circulation and worsening clinical outcomes [115,116]. Although endotoxins are typically associated with Gram-negative bacteria, recent research has shown that Gram-positive pathogens, particularly *S. aureus* and *S. pyogenes*, can induce endotoxin-like inflammatory responses. These pathogens produce superantigens, cytolysins and exotoxins, which trigger inflammation similar to that seen with LPS from Gram-negative bacteria [103,105, 117, 118]. Superantigens, for instance, can activate large populations of T-cells, leading to cytokine storms that result in systemic inflammation, organ dysfunction and septic shock [117,119, 120]. These responses mirror the effects of endotoxemia, adding complexity to the management of burn patients co-infected with SARS-CoV-2, as they are at heightened risk for hyperinflammatory reactions and immune dysregulation [121]. This effect is particularly concerning for burn patients, whose compromised immune defences increase their susceptibility to exacerbated inflammatory responses, delayed wound healing and co-infections with both Gram-positive and Gram-negative bacteria. When Gram-negative co-infections occur, the additional release of endotoxins further elevates the risk of severe complications, such as septic shock, AKI and ARDS. Recent studies highlight that in COVID-19 patients, the compounded inflammatory responses from both bacterial and viral pathogens may contribute to multi-organ dysfunction and poor patient outcomes, particularly in vulnerable groups like burn patients [105,113, 122].

### Synergistic effects of SARS-CoV-2 and bacterial toxins

Burn wounds provide a direct pathway for bacterial toxins, such as LPS and superantigens, to enter systemic circulation. These toxins exacerbate immune dysregulation by over-activating inflammatory pathways, a phenomenon particularly concerning in COVID-19 patients. Studies have shown that Gram-negative bacteria, including *A. baumannii*, *P. aeruginosa* and *E. coli*, frequently colonize the respiratory tracts of critically ill COVID-19 patients, contributing to secondary bacterial infections and worsening viral pathogenesis [11,19].

The role of bacterial endotoxins in COVID-19 severity has gained significant attention. Recent findings suggest that the SARS-CoV-2 spike protein directly interacts with LPS, amplifying immune activation and systemic inflammation. This interaction mirrors the mechanism of LPS binding to the CD14 receptor on immune cells, which triggers the release of pro-inflammatory cytokines like TNF-α, IL-1β and IL-6 [123,124]. The presence of LPS in circulation not only enhances cytokine storm severity but may also promote epithelial damage, increasing the risk of ARDS and multiorgan failure in co-infected patients [125,126].

Synergistic effects between viral and bacterial toxins extend beyond immune activation. SARS-CoV-2–mediated disruption of epithelial barriers in the respiratory and gastrointestinal tracts facilitates bacterial translocation, allowing pathogens and their toxins to access systemic circulation. This bacterial translocation aggravates systemic inflammation, compounding disease severity in COVID-19 patients with pre-existing vulnerabilities, such as burn injuries [127].

Such interactions are not unique to SARS-CoV-2. Other viral infections, such as HIV and dengue, have demonstrated similar patterns of immune hyperactivation in the presence of elevated LPS levels. These findings underscore a broader phenomenon in which bacterial toxins act as co-factors that amplify viral disease progression [111,112, 116].

Understanding the molecular mechanisms underlying these interactions is essential for identifying therapeutic targets. For instance, targeting the LPS-CD14 receptor axis or SARS-CoV-2–LPS binding pathways could help mitigate the synergistic effects of bacterial and viral infections [128,129]. These insights may guide the development of novel therapies aimed at controlling hyperinflammation and improving outcomes in burn patients co-infected with bacterial pathogens and SARS-CoV-2.

## Comparative analysis of clinical outcomes

Omranifard *et al*. carried out a retrospective observational cohort study with the goal of examining the features of adult burn patients who were hospitalized to three reference centres in Iran between October 2020 and October 2023. A total of 50.2% of patients with COVID-19 and 49.8% of patients without COVID-19 had thermal burns [130]. One noteworthy finding amongst the consequences evaluated in this study was that the COVID-19-positive cohorts had noticeably higher incidence rates of paralytic ileus and pneumonia. Similarly, almost the same study was carried out by Walters *et al*. [37], who found that patients with COVID-19 had a greater incidence of pneumonia and sepsis. Furthermore, in a cohort of burn patients, COVID-19 infection was linked to an increase in thrombosis and hypertrophic scarring but not to an increase in death. Even though their data do not show a sharp rise in mortality, this could be explained by the fact that the burn injury already activates the common pathway of IL-6, IL-8 and TNF-α activation, overshadowing the COVID-19 cytokinetic dysregulation of these cytokinetic pathways. As more information becomes available, more research is needed. These results are similarly consistent with those of El Moheb *et al*. [131], who found that COVID-19 patients frequently had more severe transaminitis, intestinal ischaemia and ileus. Furthermore, Moghimi *et al*. [132] talked about the gastrointestinal symptoms of COVID-19 and pointed out that, even in the absence of respiratory symptoms, paralytic ileus should be taken into consideration as a possible presentation of the illness.

## Therapeutic approaches for managing burn patients with co-infections during COVID-19

Managing burn patients with bacterial co-infections, particularly during the COVID-19 pandemic, requires innovative and integrated therapeutic approaches tailored to the unique challenges posed by viral and bacterial synergism. Below are some key strategies that hold promise for improving outcomes in this vulnerable population.

### Endotoxin adsorption therapy

Given the role of endotoxins in worsening bacterial and viral infection outcomes, endotoxin adsorption therapy has emerged as a promising supportive treatment for severe cases of COVID-19 pneumonia and related complications [133]. This therapy involves using devices that bind and remove circulating endotoxins from the bloodstream, effectively reducing the systemic inflammatory response and preventing endotoxin-mediated complications such as septic shock and multi-organ failure [134]. The therapy’s mechanism centres on lowering the levels of LPS, a major component of Gram-negative bacterial endotoxins, which are potent stimulators of inflammation and cytokine release. Reducing LPS levels in circulation can mitigate cytokine storm severity, which is often triggered by SARS-CoV-2 infection and exacerbated by bacterial co-infections [125].

Initial studies on endotoxin-removal devices, such as polymyxin B haemoperfusion cartridges, have shown encouraging results, demonstrating improvements in patients with sepsis and severe COVID-19 who are at risk for secondary bacterial infections [135]. Additionally, newer technologies, like resin- and charcoal-based adsorbents, are under investigation and may offer more specific endotoxin-binding capabilities with fewer side effects [136,137]. Integrating endotoxin adsorption therapy into burn care protocols, especially for COVID-19-positive burn patients with co-infections, could help manage complex inflammatory responses and reduce mortality. Future research should focus on optimizing treatment timing, understanding patient selection criteria and combining endotoxin adsorption with other supportive therapies, such as immunomodulation, to maximize patient outcomes in severe, co-infected cases [138].

### Immunomodulatory therapies

Immune dysregulation is a hallmark of both severe burn injuries and COVID-19. Managing this immune imbalance is crucial to improving outcomes in co-infected patients. IL-6 inhibitors like tocilizumab have been extensively studied for their role in reducing cytokine storms in severe COVID-19 cases. These inhibitors not only control systemic inflammation but also support the recovery of immune functionality necessary for wound healing [78,79]. JAK inhibitors, such as baricitinib, have emerged as another promising option. By modulating inflammatory pathways, JAK inhibitors can reduce cytokine overproduction whilst preserving critical immune responses against secondary bacterial infections [79].

In addition, mesenchymal stem cell (MSC) therapy is gaining attention for its dual role in immunomodulation and tissue repair. MSCs secrete bioactive molecules that dampen excessive inflammation, promote wound healing and enhance epithelial barrier function [139]. Recent trials combining MSCs with other therapies, such as endotoxin adsorption or IL-6 inhibitors, have shown potential for synergistic effects in managing complex cases of immune dysregulation [139,140].

Future directions should explore optimizing the timing of immunomodulatory interventions to balance immune suppression and activation, particularly in burn patients prone to opportunistic infections.

### Bacteriophage therapy

Bacteriophage therapy represents a highly targeted approach to addressing MDR bacteria in burn wounds. Bacteriophages are viruses that selectively infect and destroy bacteria without harming host cells. They are particularly effective against biofilm-associated bacteria such as *P. aeruginosa* and *S. aureus*, which are common in chronic burn wound infections [141,142]. Recent advancements have focused on engineering phages to enhance their efficacy and specificity. For instance, CRISPR-Cas–modified bacteriophages can target antibiotic resistance genes, preventing the emergence of further resistance [141]. Phage therapy is increasingly being explored as a complementary treatment alongside antibiotics, particularly for MDR infections where conventional therapies fail [143]. Another emerging approach is the use of phage-derived enzymes, such as lysins, which break down bacterial cell walls. These enzymes can penetrate biofilms and may offer a solution for infections in deep or chronic wounds [144]. In the context of COVID-19, bacteriophage therapy offers a unique advantage: it reduces bacterial burden without relying on broad-spectrum antibiotics, thereby minimizing the risk of further microbiota disruption. Future studies should focus on personalized phage therapy, tailored to the specific bacterial profile of each patient.

### Advanced wound care techniques

Advanced wound care technologies are transforming the management of burn wounds, particularly in patients with bacterial co-infections. Nanotechnology-based dressings, such as silver nanoparticle-coated hydrogels, have demonstrated antimicrobial and anti-inflammatory properties, making them ideal for preventing biofilm formation and promoting wound healing [8,145]. Hydrogel dressings infused with antibiotics, growth factors or immunomodulators provide controlled-release capabilities, ensuring sustained therapeutic effects whilst reducing infection risks. For deeper burns, electrospun nanofibre scaffolds infused with antimicrobial agents or growth factors can accelerate granulation tissue formation and epithelialization [143,145]. Bioengineered skin substitutes are also gaining traction. These substitutes incorporate living cells, such as keratinocytes and fibroblasts, to mimic the skin’s natural structure and function. When combined with immunomodulators or antimicrobial agents, they offer a comprehensive solution for managing severe burns complicated by infections [146]. Future research should focus on integrating wound care technologies with systemic therapies, such as bacteriophage or immunomodulatory treatments, to provide a holistic approach to burn wound management.

### Rapid diagnostics and personalized medicine

Timely and accurate diagnosis is essential for improving outcomes in burn patients with bacterial and viral co-infections, especially during the COVID-19 pandemic. Advances in diagnostic technologies are transforming clinical practice by enabling earlier identification of pathogens, resistance profiles and systemic inflammation markers, paving the way for personalized therapeutic strategies.

CRISPR-Cas technology has revolutionized diagnostics by providing a rapid, sensitive and specific method to detect pathogens and their resistance genes. CRISPR-based assays, such as Specific High-sensitivity Enzymatic Reporter unLOCKing and DNA Endonuclease-Targeted CRISPR Trans Reporter, can simultaneously identify multiple bacterial and viral pathogens in less than an hour. These tools are particularly valuable for detecting MDR bacteria like *A. baumannii* or *P. aeruginosa* in burn wounds, as well as SARS-CoV-2 in co-infected patients [145,147].

Another approach is next-generation sequencing (NGS). This technique has emerged as a comprehensive approach for profiling microbial communities in burn wounds. By sequencing bacterial, viral and fungal DNA, NGS provides detailed information about pathogen abundance, virulence factors and resistance genes. Metagenomic analysis using NGS can uncover previously undetected co-infections, enabling clinicians to tailor antimicrobial regimens more effectively [144,148]. Integration of NGS with cloud-based bioinformatics platforms can further accelerate data interpretation and decision-making.

Also, in this field, the wearable diagnostic devices and biosensors are very important. Wearable biosensors are transforming point-of-care diagnostics by enabling real-time monitoring of wound conditions. These devices can detect early signs of infection, such as changes in pH, temperature or specific biomarkers, providing clinicians with actionable insights to prevent complications. Smart bandages equipped with sensors can also track healing progress and alert healthcare providers to potential delays in recovery [146]. Future iterations of these devices may incorporate drug delivery systems, enabling localized release of antibiotics or anti-inflammatory agents based on sensor feedback.

Today, the artificial intelligence (AI) in diagnostics was very widespread. AI algorithms are being developed to analyse complex diagnostic data, such as patterns in pathogen resistance or inflammatory markers, to predict patient outcomes. Machine learning models trained on large datasets can identify high-risk patients, suggest personalized treatment plans and monitor therapy effectiveness over time. AI-powered tools have already shown promise in distinguishing between bacterial and viral infections based on clinical and laboratory data, reducing diagnostic uncertainty and unnecessary antibiotic use [149].

Rapid point-of-care platforms, such as multiplex PCR and immunoassay-based tests, allow bedside detection of pathogens and inflammatory markers in minutes. These platforms are particularly useful in resource-limited settings, where early interventions can significantly reduce mortality. Combining point-of-care diagnostics with telemedicine consultations can further enhance access to care for burn patients in remote or underserved areas [150,151].

Finally, the integration of advanced diagnostics with personalized medicine enables clinicians to tailor therapies based on a patient’s unique infection profile, immune status and wound characteristics. For instance, antimicrobial susceptibility data from rapid diagnostics can guide the selection of narrow-spectrum antibiotics, minimizing resistance development and off-target effects. Personalized immunomodulatory regimens can also be optimized to balance immune suppression and activation, reducing the risk of cytokine storms or secondary infections [152,153].

## Conclusion

One of the initial challenges in managing burn patients during the COVID-19 pandemic was the lack of standardized protocols, which led to delays in diagnosis and increased the risk of transmission to other patients and healthcare personnel. Based on accumulated experience, burn treatment centres have now adopted standardized protocols for managing burn patients requiring hospital admission. These protocols include real time PCR testing for all patients, regardless of symptoms, to prevent undetected COVID-19 cases [154]. The complexity of burn patient care remains a challenge due to the multiple physiological alterations from thermal injuries, particularly in the immune and haematologic systems. These changes can produce symptoms and laboratory findings that resemble other diseases, complicating accurate diagnoses. For example, fever due to an inflammatory response in burn patients can mask underlying infections, as healthcare providers may attribute it solely to burn trauma. Similarly, lymphopenia, a common laboratory finding in burn patients, could be misinterpreted as a sign of COVID-19, leading to delays in bacterial infection detection [155,156]. Treatment of burn patients with bacterial co-infections, especially those with MDR or extensively drug-resistant pathogens, requires specialized protocols distinct from the WHO’s recent COVID-19 guidelines. This is further complicated by the absence of specific antiviral treatments for COVID-19. Evidence suggests that cytokine storms are a primary driver of severe COVID-19, leading to conditions such as ARDS and multiorgan dysfunction, which have high mortality rates. Targeting this pathway with immunomodulatory drugs may help control severe COVID-19, especially in burn patients with bacterial co-infections [157]. In co-infected patients, a combination of immunomodulatory drugs and antibacterial agents could be beneficial. Activated MSCs induced by IFN-γ have shown promise for reducing inflammation and promoting tissue repair in severe COVID-19 cases [158,159]. Currently, the most frequently used drugs for managing severe COVID-19 are immunomodulatory agents, including IL-6 inhibitors [98,100,121, 160]. However, using these drugs in COVID-19 patients with immunosuppressed profiles, such as burn patients, must be approached carefully, as they could increase the risk of bacterial co-infections in individuals with weakened immune defences. Emerging data suggest that bacterial products may play a more significant role in triggering cytokine storms than the virus alone. Bacterial toxins and DNA found in the blood of COVID-19 patients may arise from disrupted barriers, as SARS-CoV-2 can cause capillary leakage similar to bacterial sepsis. This results in interstitial oedema and compromised lung and intestinal barriers, allowing bacterial translocation into circulation and leading to a heightened inflammatory response [125]. Therefore, further investigation into the relationship between SARS-CoV-2 and bacterial infections, as well as the mechanisms of co-infection, is critical. Understanding the interactions between viruses and bacteria could unveil new therapeutic targets and inform more effective treatment approaches. The COVID-19 pandemic has highlighted the need for specialized care and vigilant monitoring in burn patients. Timely diagnosis and rapid identification of co-infections are essential, with special attention to those who may be untreated, undertreated or asymptomatic carriers. Co-infected patients should be managed as high-priority cases, with comprehensive monitoring for both SARS-CoV-2 and bacterial pathogens [161]. Rapid diagnostic tools, including molecular-based techniques for early, on-site diagnosis and susceptibility testing, are crucial for initiating prompt, targeted treatments and improving outcomes in this vulnerable population [162]. The burn patients need healthcare and special attention, and the COVID-19 pandemic revealed its importance. Therefore, the alterations and symptoms of patients must be carefully analysed, and their diagnosis should be done as soon as possible. It is necessary to identify co-infected patients and consider them as a kind of emergency, as people who are fully or under-treated or latent should be tracked and monitored for COVID-19 disease [158]. Altogether, diagnosis and susceptible drug tests should be carried out which require rapid and on-site molecular-based techniques for early and decisive diagnosis.

## Future perspectives

Advancements in burn care during the COVID-19 pandemic underscore the need for rapid diagnostics, targeted immunotherapy and novel antimicrobial strategies to manage complex co-infections. Future research should prioritize developing point-of-care diagnostic tools that can quickly detect both viral and bacterial pathogens, particularly MDR strains. Innovations like CRISPR-based diagnostics and NGS could facilitate earlier, more accurate identification of pathogens and inform timely, individualized treatment.

Immunomodulatory therapies, such as IL-6 inhibitors, JAK inhibitors and MSCs, hold promise for managing the hyperinflammatory responses common in burn patients co-infected with SARS-CoV-2. Tailoring these therapies to individual immune profiles could help mitigate the risk of cytokine storms whilst supporting wound healing. Additionally, endotoxin-targeting treatments like adsorption therapy may help reduce systemic inflammation, providing new tools for addressing the heightened risks of co-infection.

Lastly, the integration of telemedicine and remote monitoring into burn care protocols represents a valuable advancement, especially in reducing in-person exposure risks. Remote follow-up care and virtual consultations with specialists could improve care continuity, allowing for earlier intervention in co-infected burn patients. These future perspectives collectively highlight the importance of innovation in diagnostics, therapeutics and patient monitoring to improve outcomes in burn patients during pandemics and beyond.

## Abbreviations

ACEII: angiotensin-converting enzyme II
AI: artificial intelligence
AKI: acute kidney injury
ARDS: acute respiratory distress syndrome
ICU: intensive care unit
IL: interleukin
JAK: Janus kinase
LPS: lipopolysaccharides
MDR: multidrug-resistant
MSC: mesenchymal stem cell
NGS: next-generation sequencing
RT-PCR: real time PCR
SSLs: superantigen-like toxins
TSST-1: toxic shock syndrome toxin-1
WHO: World Health Organization
